# Organic Waste Substrates Induce Important Shifts in Gut Microbiota of Black Soldier Fly (*Hermetia illucens* L.): Coexistence of Conserved, Variable, and Potential Pathogenic Microbes

**DOI:** 10.3389/fmicb.2021.635881

**Published:** 2021-02-12

**Authors:** Chrysantus M. Tanga, Jacqueline Wahura Waweru, Yosef Hamba Tola, Abel Anyega Onyoni, Fathiya M. Khamis, Sunday Ekesi, Juan C. Paredes

**Affiliations:** ^1^International Centre of Insect Physiology and Ecology (ICIPE), Nairobi, Kenya; ^2^Department of Biochemistry, Genetics, and Microbiology, Forestry and Agricultural Biotechnology Institute (FABI), University of Pretoria, Pretoria, South Africa

**Keywords:** organic waste valorization, microbiota, safety, dysbiosis, feed industries, foodborne diseases

## Abstract

The sustainable utilization of black soldier fly (BSF) for recycling organic waste into nutrient-rich biomass, such as high-quality protein additive, is gaining momentum, and its microbiota is thought to play important roles in these processes. Several studies have characterized the BSF gut microbiota in different substrates and locations; nonetheless, in-depth knowledge on community stability, consistency of member associations, pathogenic associations, and microbe–microbe and host–microbe interactions remains largely elusive. In this study, we characterized the bacterial and fungal communities of BSF larval gut across four untreated substrates (brewers’ spent grain, kitchen food waste, poultry manure, and rabbit manure) using 16S and ITS2 amplicon sequencing. Results demonstrated that substrate impacted larval weight gain from 30 to 100% gain differences among diets and induced an important microbial shift in the gut of BSF larvae: fungal communities were highly substrate dependent with *Pichia* being the only prevalent genus across 96% of the samples; bacterial communities also varied across diets; nonetheless, we observed six conserved bacterial members in 99.9% of our samples, namely, *Dysgonomonas*, *Morganella*, *Enterococcus*, *Pseudomonas*, *Actinomyces*, and *Providencia*. Among these, *Enterococcus* was highly correlated with other genera including *Morganella* and *Providencia*. Additionally, we showed that diets such as rabbit manure induced a dysbiosis with higher loads of the pathogenic bacteria *Campylobacter*. Together, this study provides the first comprehensive analysis of bacterial and fungal communities of BSF gut across untreated substrates and highlights conserved members, potential pathogens, and their interactions. This information will contribute to the establishment of safety measures for future processing of BSF larval meals and the creation of legislation to regulate their use in animal feeds.

## Introduction

The animal feed industry is currently facing considerable shortages in protein sources, and the situation is expected to aggravate further with human population growth and the increasing demand for meat in their diets (National Research Council, 2015; van Huis, 2013). Therefore, the urgent need for alternative protein sources to substitute the conventional expensive sources such as fishmeal and soya bean has become crucial. Insects are a promising and sustainable alternative protein source, and their applications as protein additives in animal feeds have been the subject of recent research (Rumpold and Schlüter, 2013). The black soldier fly (BSF; *Hermetia illucens* L.) larvae is an example of a high-quality edible insect whose adoption as an alternative animal feed presents several advantages. First, BSF larvae are rich in crude protein and has a well-balanced amino acid profile (particularly lysine, threonine, and methionine, which are cereal-limiting amino acids), fats, and micronutrients [iron and zinc (Zheng et al., 2013; De Smet et al., 2018; Shumo et al., 2019; Giannetto et al., 2020)]. Secondly, the use of BSF larvae as animal feed for poultry, fish, and swine has been deemed feasible due to the ease and economical production systems that depend largely on recycling organic waste into nutrient-rich biomass (Shumo et al., 2019). The utilization of organic wastes by the BSF has been considered as an excellent mitigation measure against environmental pollutants due to the high larval waste bioconversion efficiency (Müller et al., 2017; Wang and Shelomi, 2017). Thirdly, the insect is neither a pest nor a disease vector, making BSF larval production environmentally friendly, cost-effective, and safe (Diener et al., 2009; Shumo et al., 2019).

An efficient BSF larval production to satisfy market demands for alternative protein is nonetheless dependent on several factors such as the composition of the feeding substrates, conversion efficiency, and environmental conditions [e.g., temperature and humidity (Tomberlin et al., 2009; Wang and Shelomi, 2017)]. As such, many research efforts have focused on characterizing the nutrients and micronutrient assimilation by BSF larvae in response to different rearing conditions and substrates, as a step to optimize their yield and quality (Gold et al., 2018; Lalander et al., 2019; Shumo et al., 2019; Chia et al., 2020). Remarkably, very little research has been done on their gut microbiota and its role in BSF bioconversion and insect physiology and health.

In many insect species, symbionts—beneficial associated microbes—play crucial roles in host physiology, including nutrition, digestion, and immunity (Dillon and Dillon, 2004; Engel and Moran, 2013). Understanding the role of microbial communities in BSF has proven to be difficult since gut microbiota shifts importantly across diets and locations. Nonetheless, some evidence suggests that the gut microbiota plays important roles in larval biomass digestion (Jeon et al., 2011; Boccazzi et al., 2017; Bruno et al., 2019; Wynants et al., 2019; Klammsteiner et al., 2020). For instance, *Dysgonomonas* is thought to be involved in complex polysaccharide degradation, *Bacteroides* and *Parabacteroides* promote glycan metabolism, and *Actinomyces* facilitates the degradation of lignin and chitin (Yu et al., 2011; Zheng et al., 2013; Bruno et al., 2019; Jiang et al., 2019; Klammsteiner et al., 2020). Additionally, BSF diet supplementation with strains of gut bacteria such as *Bacillus subtilis* has been shown to promote larval growth. Studies have also reported antimicrobial properties of BSF gut microbes. These are of great interest since they might be able to reduce the load of harmful and undesired pathogens for humans in microbe-rich BSF substrates, such as manure (Erickson et al., 2004; Liu et al., 2008). For instance, it has been shown that *Trichosporon asahii* inhibits the growth of *Candida* species, most likely by the production of fungicidal molecules (Yu et al., 2011; Boccazzi et al., 2017). Other studies have shown that BSF larvae can effectively reduce *Escherichia coli* O157:H7, *Salmonella enterica* serovar enteritidis, and viruses in organic waste (Erickson et al., 2004; Lalander et al., 2014). It is therefore anticipated that pathogen waste reduction might be achieved by microbe ingestion and lysis in the gut and/or secretion of antimicrobial compounds directly into the substrate. In both processes, BSF gut microbiota might play crucial roles (Erickson et al., 2004).

Whereas the antimicrobial properties of BSF and its gut microbiota could lead to alternative valorization of the BSF system, several studies have reported as well that BSF can be a reservoirs of many significant foodborne pathogens that are detrimental to humans and animals (Bruno et al., 2019; Khamis et al., 2020). This is especially complex, when potential animal/human pathogenic bacteria associated with BSF are beneficial to the insect physiology. For example, *Providencia* in BSF is a vertically transmitted bacterium that enhances oviposition, while in humans, it causes gastroenteritis, urinary tract infections, and other nosocomial infections in immunocompromised patients (Galac and Lazzaro, 2011; De Smet et al., 2018). Other bacteria with important biosafety considerations include *Wohlfahrtiimonas*, which has been shown to cause sepsis upon myiasis infestation in two patients (Tóth et al., 2008; Almuzara et al., 2011; Kõljalg et al., 2015).

Therefore, in order to strategize ways to improve insect production, health, and safety, it is important to further characterize (i) BSF gut microbiota when reared on different substrates; (ii) identify potential conserved beneficial microbes; (iii) list and catalog potential animal and human pathogens harbored by larvae; and (iv) understand the nature of the interactions between beneficial microbes and BSF and between beneficial microbes and their pathogenic counterpart.

In this study, we characterized the gut microbiota (bacterial and fungal communities) of BSF larvae fed on four different substrates, brewers’ spent grains, kitchen food waste, poultry manure, and rabbit manure, to evaluate the impact of the different substrates on microbiota shifts as well as the promotion or absence of potential pathogenic microbes. Further, we provide insights on microbial diversity, their consistency in the gut of BSF across substrates, and microbe–microbe interactions as a step toward understanding the role of the gut microbiota in insect physiology and health (Klammsteiner et al., 2020).

## Materials and Methods

### BSF Larval Sample Collection and Preparation

Fifth-instar BSF larvae fed on four different substrates, brewers’ spent grains, kitchen food waste, poultry manure, and rabbit manure, were collected from the Animal Rearing and Containment Unit (ARCU) at the International Centre for Insect Physiology and Ecology (*icipe*, Nairobi, Kenya). The brewers’ spent grains used were sourced from the Kenya Breweries Limited, Nairobi, Kenya. Brewers’ spent grains are the common substrate used at *icipe* to rear BSF larvae, and they were considered as the “control” substrate. On brewers’ spent grains, the BSF larvae successfully complete their development in 16–21 days (Chia et al., 2018). The substrate has also been shown to promote high nutritional quality of the larvae (Meneguz et al., 2018; Chia et al., 2020). Samples of BSF larvae from the various rearing substrates were collected after 14 days and washed in 40% bleach, followed by 70% ethanol and finally 1 × PBS for 2 min to eliminate any external microorganism or contaminant DNA attached to the cuticle. Twenty entire guts per substrate were then dissected aseptically using forceps, and each gut was placed in a 2-ml microcentrifuge tube containing 750 μl CTAB solution (20 g CTAB in 100 ml CTAB base: 100 ml 1 M Tris-HCL pH 8, 280 ml 5 M NaCl, 40 ml 0.5 M EDTA pH 8, complete with Milli-Q H_2_O to a liter), 2 μl beta-mercaptoethanol, and 100–200 μl beads (3 mm diameter). Samples were then stored at −80°C until DNA extraction.

### DNA Extraction

The CTAB–phenol–chloroform DNA extraction method was used in this study adapted from Ausubel et al. (1988). Briefly, freeze-thawed gut samples (see described above) were homogenized using the QIAGEN TissueLyser II, and 1 ml of phenol was added, mixed vigorously for 10 s, and incubated at 64°C for 6 min. The CTAB/phenol homogenate was then put in a fresh 2-ml tube containing 400 μl of chloroform and mixed by inverting several times. Centrifugation at 13,000 rpm for 10 min at room temperature followed, and the aqueous layer was transferred into a new microcentrifuge tube, to which 500 μl of phenol–chloroform–isoamyl alcohol in the ratio of 25:24:1, respectively, was added. Contents in the tubes were then mixed and centrifuged at 13,000 rpm for 3 min at room temperature, and the aqueous layer in each tube was again transferred into a new microcentrifuge tube. Five hundred microliters of chloroform was then added to each tube, and then centrifugation at 13,000 rpm for 3 min at room temperature followed. Next, the resulting aqueous layer was transferred into a new microcentrifuge tube, and the DNA was precipitated by adding 900 μl of absolute ethanol and incubated overnight at −80°C. Precipitated DNA was then chilled on ice, followed by centrifugation at 13,000 rpm for 30 min at 4°C, resulting in a DNA pellet. The DNA pellet was washed twice with 1 ml of 70% ethanol and then dried at room temperature for 10 min. Lastly, the DNA was resuspended in 200 μl sterile water, and DNA concentration per sample was confirmed using NanoDrop (Thermo Scientific).

### 16S rRNA and ITS Gene Amplification and Sequencing

The DNA from 20 individual guts per substrate was shipped to Macrogen Europe BV (Meibergdreef, Amsterdam, Netherlands) for 16S rRNA (10 samples from 10 individual guts) and ITS (10 samples from 10 individual guts) region amplification and sequencing using the Illumina MiSeq platform. The V3–V4 region of the 16S rRNA was amplified using the 314F-CCTACGGGNGGCWGCAG and 805R-GACTACHVGGGTATCTAATCC primers while F5-GCATCGATGAAGAACGCAGC and R5-TCCTCCGCTTATTGATATGC were used to amplify the ITS2 region. Whereas poultry-fed larval samples followed the same treatment procedures during their DNA extraction and had comparable DNA starting material, ITS2 amplicons from these samples did not meet the DNA quantity and quality required for library preparation, three times. This was potentially due to low loads of fungi in poultry-fed larval samples.

### Data Analysis

Paired-end sequences spanning the V3–V4 and ITS2 regions of the bacterial 16S rRNA and fungi, respectively, were analyzed using QIIME 2 (version 2020.2). Briefly, the reads were imported into QIIME 2, followed by quality checking, and all the present adapters and primers trimmed using Cutadapt (version 1.8) embedded in QIIME 2. Subsequently, low-quality bases were trimmed, the forward and reverse reads merged, and chimeras removed using the DADA2 pipeline in QIIME 2. Taxonomic classification was done against the SILVA 132 database using a pre-trained naïve Bayes classifier in the case of bacteria and the UNITE (version 7-99) database in fungi.

The taxonomy file and sequences were then imported into R for downstream analysis. We first filtered unwanted sequences, taxonomies, and features including those of chloroplast, Eukaryota, and Archaea and those that were unassigned to any taxonomic group by the SILVA database after reading the three files into R. Sequences not classified up to the genus level were extracted, and a BLAST search was performed to classify them. The taxonomizr package in R was used to complete the different taxonomic classification levels. The BLAST taxonomic classification results were combined with those from SILVA 132, the taxonomy table was merged to the abundance table, and a relative abundance barplot was plotted in R (Supplementary Table S1).

Next, we rarefied the data (Supplementary Figure S1) to even sampling depths and performed alpha diversity estimation to determine richness using chao1 and Faith’s phylogenetic diversity, and diversity was determined using the Shannon diversity index with all the samples grouped according to the sample metadata. The statistical significance differences of gut microbiota richness and diversity across the different substrates were tested using the Kruskal–Wallis *H*-test (Vargha et al., 1998). Beta diversity estimations on the other hand were done using Venn diagrams and weighted and unweighted UniFrac distance metrics at the genus level (Lozupone and Knight, 2005). PERMANOVA was done to compare microbial communities across different substrates. Lastly, the microbial abundance interaction among the most abundant microbiota was established using an abundance correlation-based network generated in R using the Psych package, to establish how the most abundant bacteria in the gut of BSF larvae interacted. This was also done to elucidate whether there are any bacteria in the gut of BSF larvae that influence the presence or abundance of others, especially potential pathogens.

## Results

### BSF Larval Weight Gain in Four Untreated Substrates

**FIGURE 1:**
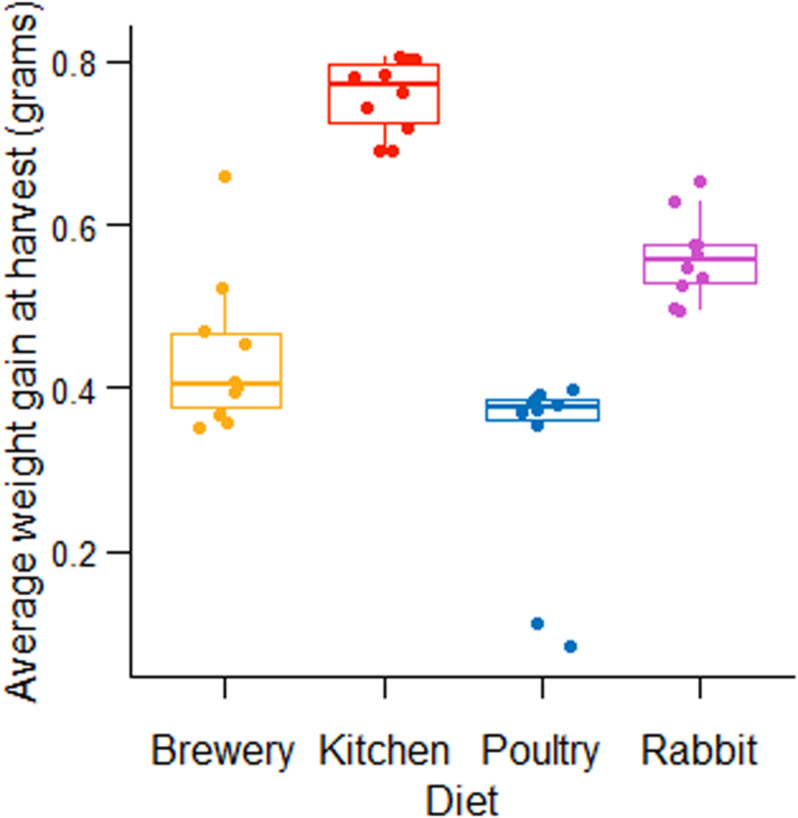
Fifth instar BSF larval weight fed on four untreated diets.

We started by measuring the weight gain of BSF larvae in different substrates. We observed that the substrate that promoted the highest BSF neonate (5 days old, fifth-instar larvae) growth was kitchen waste with a larval weight of 0.76 g, followed by rabbit manure (0.56 g weight per larva), brewers’ spent grains (0.44 g per larva), and chicken manure (0.32 g per larva) (Figure 1).

### Bacterial Communities Associated With BSF Guts

**FIGURE 2:**
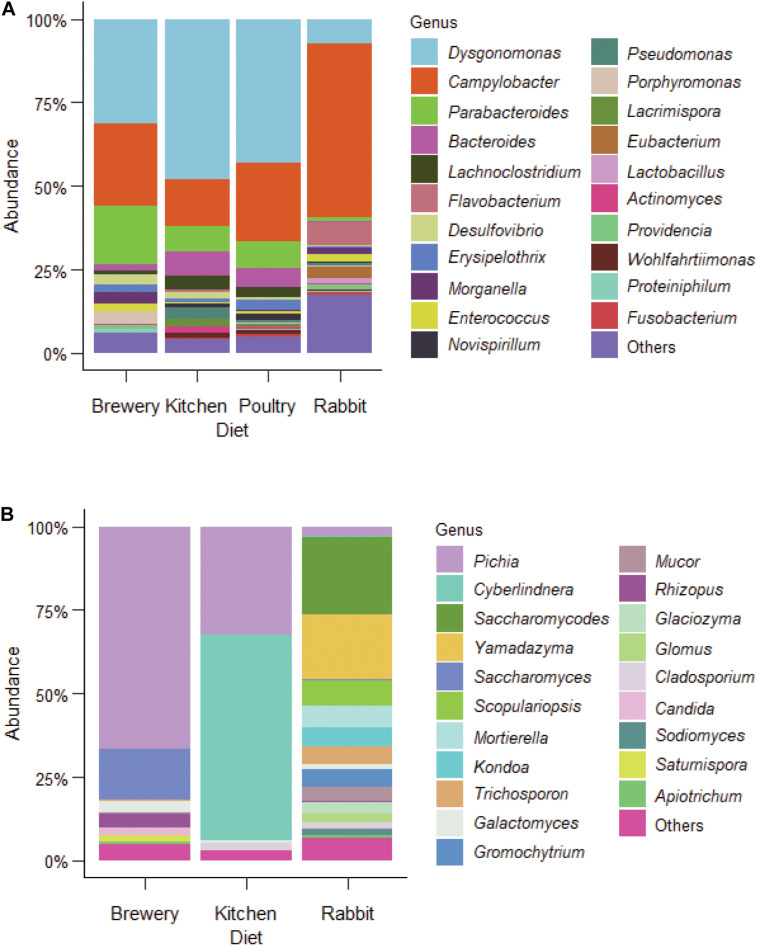
Bacterial and fungal microbiota profiles in BSF larvae fed on different diets. **(A)** bacterial and **(B)**, fungal microbial profile from BSF larvae guts fed on brewers’ spent grain, kitchen food waste, poultry manure, and rabbit manure.

In order to evaluate the role of the gut microbiota in BSF growth and uncover potential shifts in BSF gut microbiota induced by the diet, we characterized bacterial and fungal communities of the BSF gut. Out of the 39 BSF larval gut samples profiled in this study from four different substrates [brewers’ spent grains, kitchen waste (food waste), poultry manure, and rabbit manure], we recovered a total of 4,665,023 paired-end sequences spanning the V3–V4 region of the bacterial 16S rRNA. By analyzing all the sequenced reads, we identified 3,282 amplicon sequence variants (ASVs). From our analysis, we characterized 21 genera, with an overall relative abundance higher than 0.5%, as BSF representative gut bacterial communities (Figure 2A, Supplementary Figure S2A and Supplementary Table S2). From our dataset, the dominant phylum was Bacteroidetes with the most abundant genera being *Dysgonomonas*, *Parabacteroides*, *Bacteroides*, and *Flavobacterium*. *Dysgonomonas* alone accounted for about 32% of total reads. The second was Proteobacteria, which included *Campylobacter*, *Desulfovibrio*, and *Morganella*, with *Campylobacter* alone accounting for about 27% of total reads. Firmicutes was the third phylum including *Lachnoclostridium*, *Erysipelothrix*, and *Enterococcus*. We also uncovered *Actinomyces*, *Providencia*, and *Wohlfahrtiimonas*, but with low relative abundance (>1%, Figure 2A). Among all the identified genera in this study, *Dysgonomonas*, *Campylobacter*, *Desulfovibrio*, *Erysipelothrix*, *Morganella*, *Enterococcus*, *Pseudomonas, Actinomyces*, and *Providencia* were among the most prevalent across samples with 99.9% of prevalence (Supplementary Table S1).

### Substrate Effect on Bacterial Communities

**FIGURE 3:**
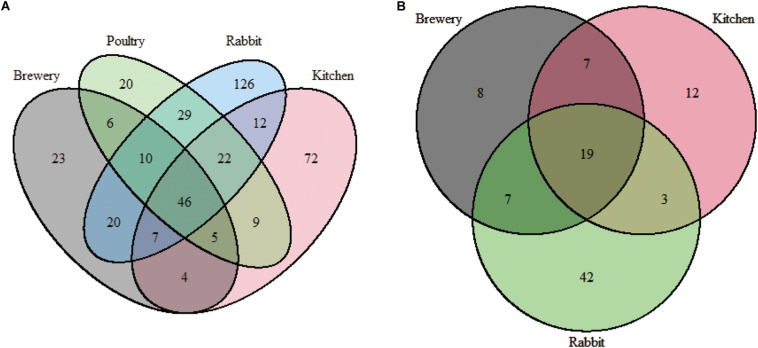
Shared bacterial and fungal gut diversity in BSF larvae fed on different diets. **(A)** bacteria, **(B)** fungi.

**FIGURE 4:**
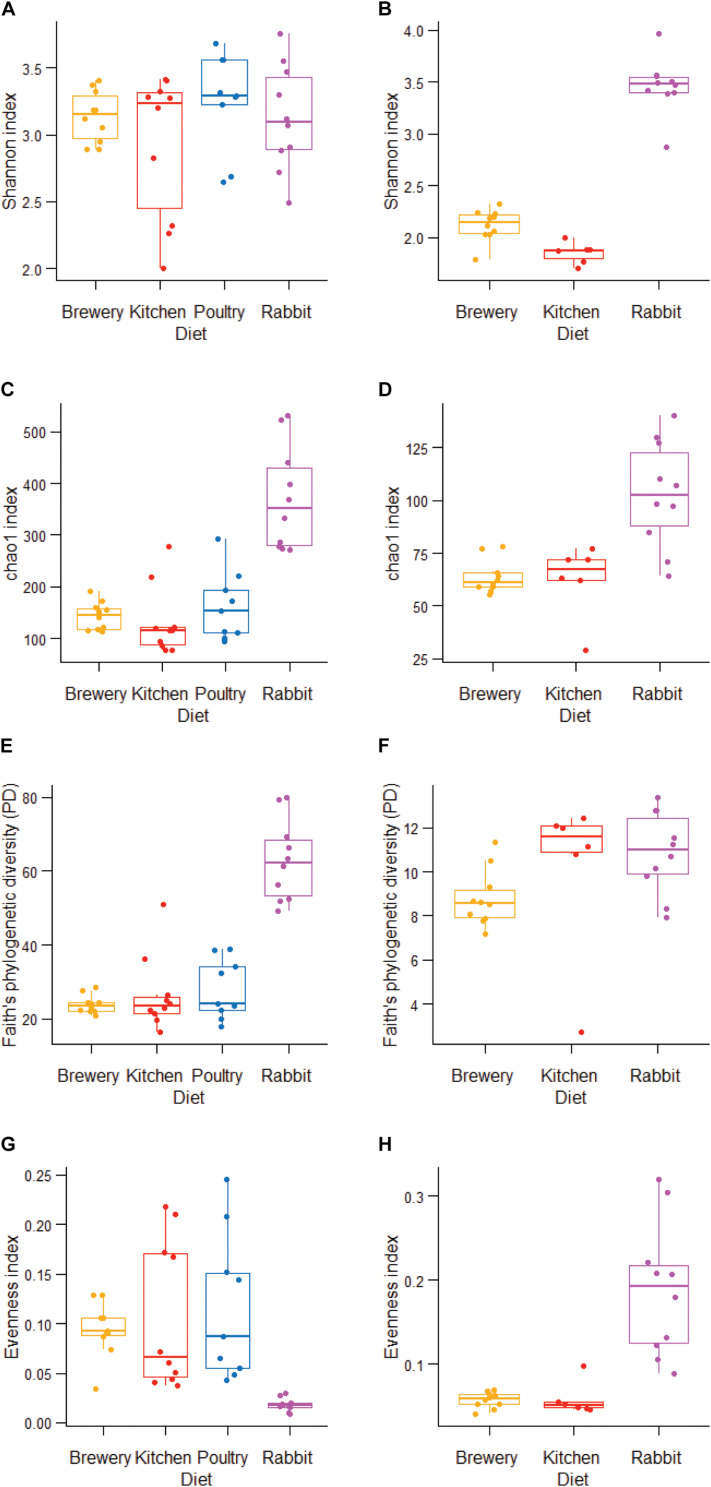
Alpha diversity in bacterial and fungal gut communities in BSF larvae fed on different diets. **(A,B)** *Shannon* diversity index estimates, **(C,D)** *chao1* richness index estimates. **(E,F)** *Faith’s Diversity* estimates. **(G,H)** *Evenness* estimates, from bacterial **(A,C,E,G)** and fungal **(B,D,F,H)** communities.

**FIGURE 5:**
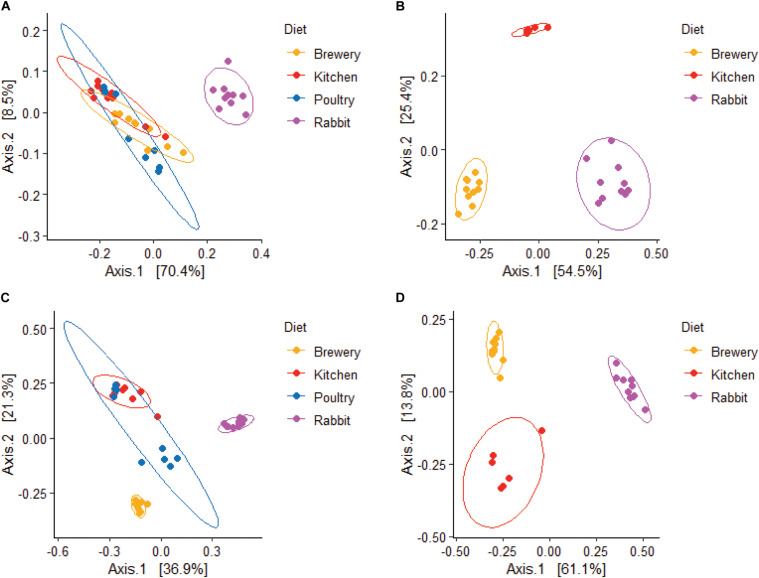
Weighted and Unweighted unifrac distance estimates from gut communities in BSF larvae fed on different diets. Weighted **(A,B)**, and Unweighted **(C,D)**, unifrac distance estimates from bacterial **(A,C)**, and fungal **(B,D)**, communities.

Forty-six bacterial genera were common across all substrate types. Rabbit manure harbored 126 unique bacterial genera, followed by chicken manure with 72 unique genera (Figure 3A). We observed that the alpha diversity in BSF guts varies considerably when richness (*chao1*: *p* = 0.0001, Figure 4C), phylogenetic distance (*Faith’s phylogenetic diversity*, *p* = 0.000073, Figure 4E), and evenness (*evenness*: *p* = 0.00007, Figure 4G) were tested. We found that in these three metrics, the rabbit manure substrate was clearly set apart from the other three substrates. These results are consistent with the principal coordinate analysis (PCoA) using both unweighted and weighted UniFrac (Figures 5A,C). Larvae fed on kitchen waste and poultry manure showed bacterial communities that clustered together in both weighted and unweighted UniFrac, whereas brewers’ spent grain clustered close to those fed on poultry manure using weighted but not unweighted UniFrac analysis (Figures 5A,C). Furthermore, the substrate effect was further confirmed by pairwise PERMANOVA, where substrate had a significant impact on gut bacterial communities with (*R*^2^ = 0.47485, *p* = 0.01, Supplementary Table S3A). Jointly, the four substrates in this study had a significant impact on the gut bacterial communities of BSF larvae with rabbit waste having the most severe effect.

### Fungal Communities Associated With BSF Guts

From 26 samples, we characterized the BSF gut fungal communities from larvae reared on three different substrates: brewers’ spent grains, kitchen waste, and rabbit manure. A total of 3,840,437 paired-end sequences spanning the fungal ITS2 region were analyzed, and 654 fungal ASVs were identified, 277 of which were classified as yeast representing 69.2% of total reads. A total of 20 fungal genera with a relative abundance greater than 0.5% were presented in Figure 2B. The dominant genera were *Pichia*, *Cyberlindnera*, *Saccharomycodes*, *Yamadazyma*, *Saccharomyces*, and *Scopulariopsis*. At the species level, *Pichia kudriavzevii* dominated in the brewers’ spent grain and rabbit manure (25.8%) with the highest prevalence (96%), while *Cyberlindnera jadinii* was dominant in BSF larvae fed on kitchen waste (15.3%, Supplementary Figure S2B). A total of 19 genera were common across substrates with their cumulative relative abundance representing 60.8% of total reads (Figure 3B and Supplementary Table S1).

### Substrate Effect on Fungal Communities

We found a significant impact of diets on fungal microbiota richness, diversity, and evenness (Shannon, *p* = 0.00003; *chao1*, *p* = 0.0013; *Faith’s phylogenetic diversity*, *p* = 0.023; *evenness*, *p* = 0.00015; Figures 4B,D,F,H) across substrates, especially when larvae were fed on rabbit manure. The dichotomy among substrates observed in Figures 2–4 was further confirmed by beta diversity analysis. Unweighted and weighted UniFrac analysis (Figures 5B,D) and PERMANOVA pairwise analysis (Supplementary Table S3B, *R*^2^ = 0.81352, *p* = 0.001) showed that the fungal communities in BSF guts clustered by substrate. Together, the substrate had a severe impact on the fungal community’s composition and abundance in BSF larval gut.

### Gut Microbiota Member Interactions

**FIGURE 6:**
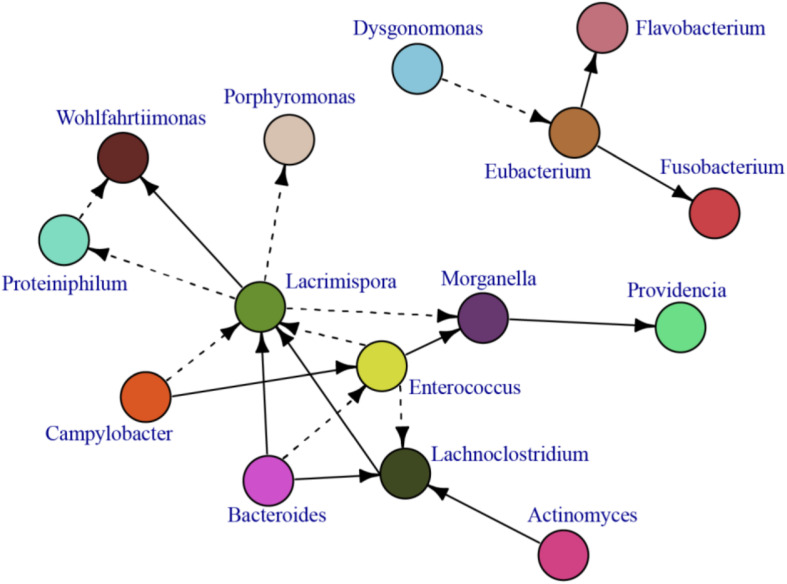
A correlation network demonstrating the BSF gut bacterial interactions. A cut of 0.65 and above was used to distinguish the most significant correlation.

In our analysis, we found several pathogenic opportunistic bacteria including *Campylobacter*, *Morganella*, *Wohlfahrtiimonas*, and *Providencia* (Figure 2A and Supplementary Figure S2A) and opportunistic fungus teleomorph (the sexual reproduction stage) *Candida* species (e.g., *Cyberlindnera* sp. and *Trichosporon* sp.) (Figure 2B and Supplementary Figure S2B). The relationship between potential bacterial pathogens and the rest of the gut bacterial microbiota is presented in an interaction network based on the correlation of the most abundant bacterial microbiota of BSF larvae (Figure 6). Among bacterial communities, *Enterococcus*, *Bacteroides*, and *Campylobacter* had numerous interactions with other bacterial species. *Enterococcus* was positively correlated with *Morganella*, which in turn was positively correlated to *Providencia*. Surprisingly, *Dysgonomonas*, the most dominant genus, did not interact with the other postulated core microbiota genera and only negatively correlated with *Eubacterium*. A similar pattern was found for *Actinomyces*, which only positively correlated with *Lachnoclostridium* (Figure 6).

## Discussion

In this study, we characterized the bacterial and fungal communities of the BSF larval guts raised in four different substrates. We observed that the substrate affected both the bacterial and fungal communities in BSF, but the effect was more severe in fungal microbiota. A close examination of the bacterial microbiota in individual samples revealed that some genera, reported in other studies, were present in almost all our samples (99.9%) despite the substrate, pointing toward a potential stable resident core gut microbiota in BSF. Additionally, we uncovered that the substrate can induce an important bacterial shift leading sometimes to dysbiosis, where one pathogenic bacterium dominated the gut microbiota without detrimental effect on the insect’s growth performance and health. Finally, we established a microbiota network in an attempt to better understand microbe–microbe interactions and guide microbe-based strategies to improve insect production, health, and safety.

We uncovered previously reported genera in great abundance such as *Dysgonomonas*, *Morganella*, *Enterococcus*, *Pseudomonas*, *Actinomyces*, and *Providencia*, genera that have been postulated as the BSF core gut microbiota members by Klammsteiner et al. (2020). In their study, Klammsteiner et al. (2020) reported that *Dysgonomonas*, *Actinomyces*, and *Enterococcus* accounted for 44% of the total number of reads with 80% prevalence across all the samples. In this study, the three accounted for a similar abundance, 34.1% of total reads, while their prevalence was much higher, 99.9% across all the samples. *Dysgonomonas* has been reported to be always among the top three most abundant members (Bruno et al., 2019; Khamis et al., 2020; Klammsteiner et al., 2020), and in our study, it accounted for 32% of the reads. Whereas the prevalence of *Dysgonomonas*, *Morganella*, *Enterococcus*, *Pseudomonas*, *Actinomyces*, and *Providencia* across studies, substrates, and locations indicates that they are conserved members of the BSF larval gut microbiota, the evidence that they play positive interactions among them and with their host, and whether they have been associated with their host over evolutionary timescales, has not been studied. This remains crucial in order to determine if the BSF larva has a core gut microbiota and if these genera could be cataloged as such.

While *Campylobacter* have been found in other studies, they have never been reported as abundant across samples as in this study [27% of total reads (Wynants et al., 2019; Klammsteiner et al., 2020)]. Their dominance is even greater in rabbit manure where they account for more than 50% of total reads. *Campylobacter* are gastrointestinal pathogens that cause diarrhea in humans and are considered as opportunistic bacteria that are usually self-limiting. They can infect a range of animals including most food production animals (Blaser, 1997). Since the most common source of *Campylobacter* infection for humans is food and water, it is particularly important to evaluate their survival along the BSF food chain (BSF larval processing, storage, BSF fed animal gut tract, and BSF fed animal meat) in order to evaluate their real potential risk and safety. It is noteworthy that *Campylobacter* might be seasonal or breeding place dependent since Khamis et al. (2020) did not report *Campylobacter* among the most abundant genera found in wild samples across the world but more importantly in Kenya. Additionally, rabbit manure-fed larvae presented the highest loads of *Campylobacter*; thus, we speculate that rabbit manure might be the source of contamination since rabbit manure is used in our rearing facility as an attractant for ovipositing flies in all substrates. However, further analysis of rabbit manure and egg chorion-associated bacteria should be performed to confirm its potential as source of contamination.

In addition to already reported genera, we found specific bacteria never described before in BSF gut such as *Lachnoclostridium*, *Flavobacteria*, *Desulfovibrio*, *Eubacteria*, and *Proteiniphilum* (Jeon et al., 2011; Zheng et al., 2013; Bruno et al., 2019; Wynants et al., 2019; Khamis et al., 2020; Klammsteiner et al., 2020). We speculate that these specific genera proliferated in BSF substrates as they are not found consistently in every study, suggesting that they might be acquired during feeding and transit through the gut without particular selection or retention in the host (Kolton et al., 2016; Tomazetto et al., 2018; Saha et al., 2020). This aspect together with the fact that no consistent subset of OTUs, besides the postulated core microbiota, are found among studies highlights that most of the bacteria found in the BSF gut are transient and are affected by the local environment.

While the substrate affects the diversity and abundance of bacterial and fungal communities found in BSF larval gut, it could lead to extreme cases of dysbiosis. Bruno et al. (2019) previously reported that an unbalanced substrate (fish based substrate) promotes Proteobacteria, mainly *Providencia*, microbiota dominance. In this study, rabbit manure also promotes Proteobacteria dominance, although in our case, it is *Campylobacter* (and in a minor extent, *Flavobacterium* from the Bacteroidetes phylum) but not *Providencia*. Whereas in Bruno et al. (2019), an unbalanced substrate reduced BSF performance, in our case, overproliferation of *Campylobacter* did not compromise larval growth and survival. This draws special attention to the fact that even if BSF is omnivorous, specific consideration has to be taken, particularly with the safety quality of the substrates, especially when BSF larvae performed well in such substrates. Although specific substrates might not directly or indirectly affect the performance and/or safety of BSF larvae, the economic value and safety of BSF larval meal (BSFLM) for use in animal feeds remains a major concern to be addressed.

Bacterial interaction analysis showed limited interactions (correlations in abundance) among all bacteria, but particularly few among the postulated core gut microbiota genera. *Enterococcus*, one of the most interconnected, positively correlated with *Morganella* and negatively correlated with *Lacrimospora* and *Lachnoclostridium*, and *Morganella* positively correlated with *Providencia*. Surprisingly, *Dysgonomonas* and *Actinomyces* showed poor interactions with others; *Dysgonomonas* negatively correlated only with *Eubacterium* and *Actinomyces* and positively correlated only with *Lachnoclostridium*. *Campylobacter*, the most dominant opportunistic clinical pathogen in this study, positively correlated with *Enterococcus* and *Lacrimospora*. Whereas analysis of more samples are needed to get a robust correlation network, these results give hints about the central role of *Enterococcus* modulating *Morganella* and *Providencia* abundance.

Mycobiome analysis in this study revealed that the fungal community in BSF guts was entirely substrate dependent as was also observed by Boccazzi et al. (2017). We found that only *Pichia* presented a high relative abundance and prevailed across all the substrates; the rest of the identified fungal communities were highly substrate specific. The high prevalence and dominance of *Pichia* in this study as well as in that of Boccazzi et al. (2017) points toward a stable association with BSF larval gut. *P. kudriavzevii*, the most prevalent species found in our samples, has been reported to encode the antibacterial toxin RY55 that is active against several human pathogens such as *E. coli*, *Enterococcus faecalis*, *Klebsiella* sp., *Staphylococcus aureus*, *Pseudomonas aeruginosa*, and *Pseudomonas alcaligenes* (Boccazzi et al., 2017). Despite the fact that the mycobiome of BSF larvae depends on the substrate, their antimicrobial activities and ability to colonize the BSF gut open an important potential for substrate supplementation and, thus, increase BSFLM safety for its use for food and feed.

We conclude that whereas bacterial and fungal communities in the BSF larval gut varied greatly across substrates, few members remained constantly associated across substrates, pointing toward a potential ecologically distinctive core gut microbiota in BSF larvae. The presence of potentially opportunistic pathogens in the gut of freshly harvested BSF larvae from large-scale production systems underpins the need of pretreatment of substrates to minimize risk of pathogen contamination along the insect-based feed value chain. The hurdles that need to be overcome in order to introduce BSFLM as a viable protein additive option in animal feed include safety concerns and product applications. Results indicate that postharvest processes such as steaming, blanching, and drying would be important and effective tools to increase microbial safety. Further research into the kinds of substrates as well as the processing and storage parameters is required to ensure that BSFLM fulfills global food safety criteria. This information will feed directly into creating legislation to regulate the use of BSFLM for animal consumption, which will in turn make its utilization at industrial scale more attractive.

## Data Availability Statement

The datasets presented in this study can be found in online repositories. The names of the repository/repositories and accession number(s) can be found in the article/Supplementary Material.

## Author Contributions

JP, CT, FK, and SE: conceptualization. JW, YT, and AO: investigation. JW and YT: data curation. JW and JP: formal analysis, visualization and writing—original draft preparation. CT, FK, and SE: funding acquisition. JW, YT, and JP: methodology. JP, CT, and FK: writing—review and editing. CT: project administration. All authors contributed to the article and approved the submitted version.

## Conflict of Interest

The authors declare that the research was conducted in the absence of any commercial or financial relationships that could be construed as a potential conflict of interest.

## Supplementary Material

Supplementary Figure 1 Bacterial rarefaction curve **(A)**, and fungal rarefaction curve **(B)**. Bacterial reads were rarefied to an even depth of 34750 while fungal reads were rarefied to an even depth of 78365.

Supplementary Figure 2 **(A)** Bacteriaand, **(B)** fungal gut communities at the species level in BSF larvae fed on different substrates.

Supplementary Table 1 16S rRNA and ITS sequencing data analysis.

Supplementary Table 2 Relative abundance of the most prevalent bacteria **(A)**, and fungi **(B)**.

Supplementary Table 3 PERMANOVA pairwise analysis from the BSF guts. Bacterial community’s pairwise analyses are presented in **(A)**, while **(B)**, represents the analysis from the fungal communities.
