# Supplementary figures and images for: Organic Waste Substrates Induce Important Shifts in Gut Microbiota of Black Soldier Fly (Hermetia illucens L.): Coexistence of Conserved, Variable, and Potential Pathogenic Microbes
Source: Front Microbiol. 2021 Feb 12;12:635881. doi: 10.3389/fmicb.2021.635881 (PMC7907179; doi:10.3389/fmicb.2021.635881)

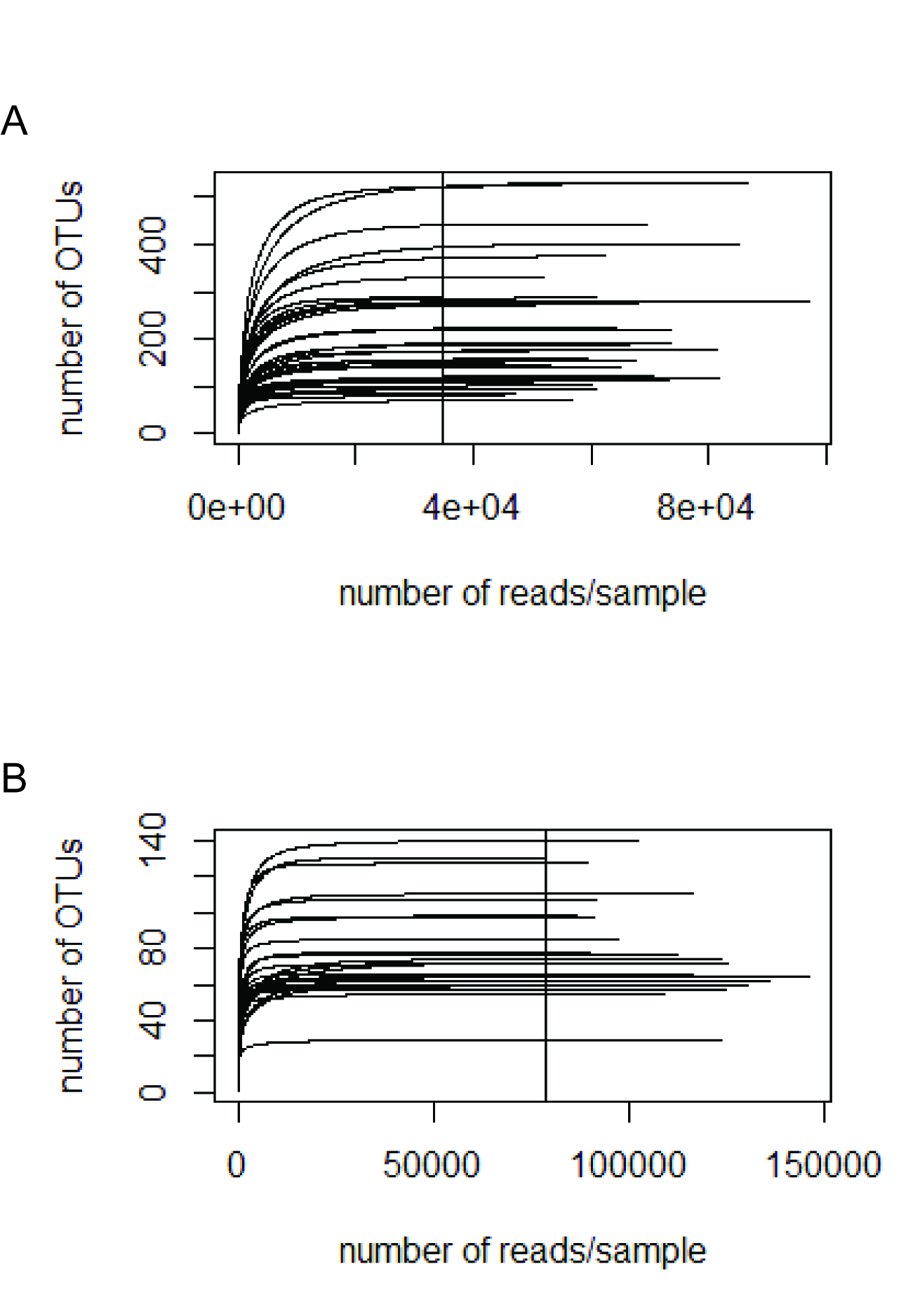

Supplement: Supplementary Figure 1 — Bacterial rarefaction curve (A), and fungal rarefaction curve (B). Bacterial reads were rarefied to an even depth of 34750 while fungal reads were rarefied to an even depth of 78365. [file Image_1.tif]

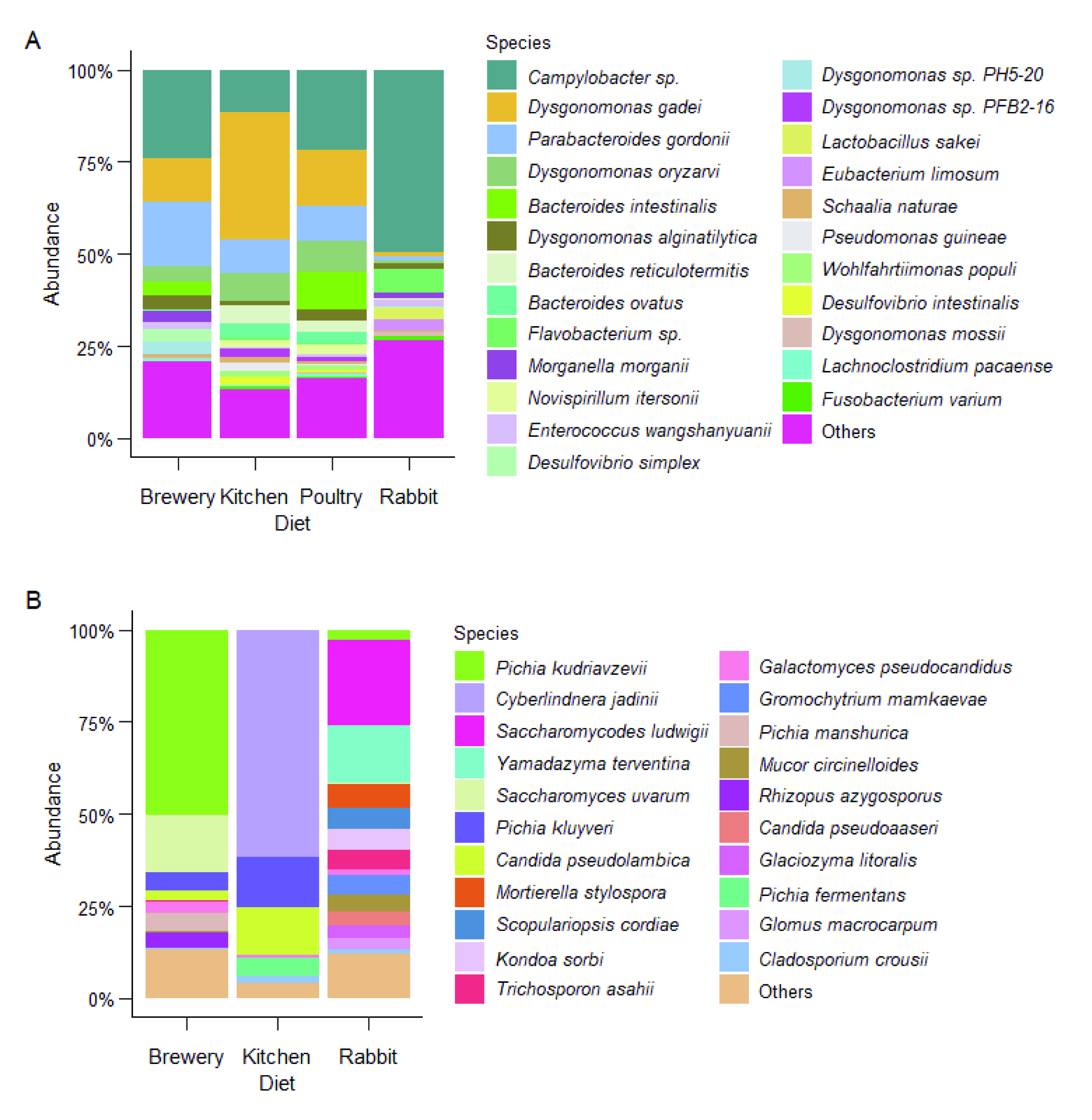

Supplement: Supplementary Figure 2 — (A) Bacteriaand, (B) fungal gut communities at the species level in BSF larvae fed on different substrates. [file Image_2.tif]
