# Supplementary material for: Organic Waste Substrates Induce Important Shifts in Gut Microbiota of Black Soldier Fly (Hermetia illucens L.): Coexistence of Conserved, Variable, and Potential Pathogenic Microbes
Source: Front Microbiol. 2021 Feb 12;12:635881. doi: 10.3389/fmicb.2021.635881 (PMC7907179; doi:10.3389/fmicb.2021.635881)
Supplement: Supplementary Table 2 — Relative abundance of the most prevalent bacteria (A), and fungi (B). [file Table_2.pdf]

Table S2. Relative abundance of the most prevalent bacteria (A) and fungi (B).

**Bacteria**	**Relative abundance (%)**
Dysgonomonas	32
Campylobacter	27
Erysipelothrix	1.65
Desulfovibrio	1.6
Morganella	1.5
Enterococcus	1.3
Pseudomonas	1.2
Actinomyces	0.8
Providencia	0.75
