# Supplementary material for: Organic Waste Substrates Induce Important Shifts in Gut Microbiota of Black Soldier Fly (Hermetia illucens L.): Coexistence of Conserved, Variable, and Potential Pathogenic Microbes
Source: Front Microbiol. 2021 Feb 12;12:635881. doi: 10.3389/fmicb.2021.635881 (PMC7907179; doi:10.3389/fmicb.2021.635881)
Supplement: Supplementary Table 3 — PERMANOVA pairwise analysis from the BSF guts. Bacterial community’s pairwise analyses are presented in (A), while (B), represents the analysis from the fungal communities. [file Table_3.pdf]

Table S3. PERMANOVA pairwise analysis from the BSF guts. Bacterial community pairwise analysis are presented in (**A**), while (**B**) represents the analysis from the fungal communities.

A

**Pairs**	R^2	***p*. value**
Brewery waste vs Kitchen waste	0.4375516	0.001
Brewery waste vs Poultry waste	0.2448472	0.001
Brewery waste vs Rabbit waste	0.5257139	0.001
Kitchen waste vs Poultry waste	0.1734253	0.016
Kitchen waste vs Rabbit waste	0.4907770	0.01
Poultry waste vs Rabbit waste	0.3541034	0.001

B

**Pairs**	R^2	***p*. value**
Brewery vs Kitchen	0.9216147	0.001
Brewery vs Rabbit	0.7865690	0.001
Kitchen vs Rabbit	0.7520458	0.001
